# Protective Effects of Protopanaxatriol Saponins on Ulcerative Colitis in Mouse Based on UPLC-Q/TOF-MS Serum and Colon Metabolomics

**DOI:** 10.3390/molecules27238346

**Published:** 2022-11-30

**Authors:** Fulin Wu, Sihan Lai, Hao Feng, Juntong Liu, Dongxing Fu, Caixia Wang, Cuizhu Wang, Jinping Liu, Zhuo Li, Pingya Li

**Affiliations:** 1 School of Pharmaceutical Sciences, Jilin University, Changchun 130021, China; 2 College of Basic Medicine Sciences, Jilin University, Changchun 130021, China

**Keywords:** ulcerative colitis, protopanaxatriol saponins, anti-inflammatory activity, metabolomics

## Abstract

Ulcerative colitis (UC) is a chronic, nonspecific inflammation of the bowel that mainly affects the mucosa and submucosa of the rectum and colon. Ginsenosides are the main active ingredients in ginseng and show many therapeutic effects in anti-inflammatory diseases, cancer, and nervous system regulation. Protopanaxatriol saponin (PTS) is an important part of saponins, and there is no research on its pharmacological effects on colitis. In this study, a model of ulcerative colitis in mice was induced by having mice freely drink 3.5% dextran sodium sulfate (DSS) solution, and UPLC-Q-TOF-MS-based metabolomics methods were applied to explore the therapeutic effect and protective mechanism of PTS for treating UC. The results showed that PTS could significantly prevent colon shortening and pathological damage and alleviate abnormal changes in UC mouse physiological and biochemical parameters. Moreover, PTS intervention regulated proinflammatory cytokines such as TNF-α, IL-6, and IL-1 in serum, and MPO and NO in colon. Interestingly, PTS could significantly inhibit UC mouse metabolic dysfunction by reversing abnormal changes in 29 metabolites and regulating eleven metabolic pathways. PTS has potential application in the treatment of UC and could alleviate UC in mice by affecting riboflavin metabolism, arachidonic acid metabolism, glycerophospholipid metabolism, retinol metabolism, and steroid hormone biosynthesis and by regulating pentose and glucuronate conversion, linoleic acid metabolism, phenylalanine metabolism, ether lipid metabolism, sphingolipid metabolism, and tyrosine metabolism, which points at a direction for further research and for the development of PTS as a novel natural agent.

## 1. Introduction

Ulcerative colitis (UC) is a chronic, nonspecific inflammation of the bowel that mainly affects the mucosa and submucosa of the rectum and colon [1]. The typical clinical symptoms are abdominal pain, diarrhea, and blood in the stool [2]. With the rapid development of industrialization, UC has become a global disease with a rapidly increasing incidence. Epidemiological data have shown that the incidence and prevalence of ulcerative colitis has increased from 1.5-fold to nearly 20-fold [3]. Long-term colitis increases the risk of colorectal cancer, which has become a very large medical burden for both patients and the government [4]. However, the pathogenesis of UC is still not fully understood. It is currently thought to be related to geography, age, gender, genetics, and environment [5]. Although there are many treatments for ulcerative colitis, serious side effects and incomplete cures have not yet been overcome. Therefore, it is urgent to screen drugs with higher efficacy and less side effects.

Ginseng (Panax ginseng C. A. Meyer) is widely used as a traditional medicine and continues to enjoy worldwide popularity for its medicinal properties. In traditional Chinese medicine, ginseng is a well-known key herb for tonifying Qi and strengthening the spleen [6]. It is used to treat a number of illnesses, including diabetes, depression, and inflammation [7,8,9], and many studies have shown that ginseng can regulate the intestinal microbiota and treat colitis [10,11,12]. Ginsenosides are the main active ingredients and show many therapeutic effects in anti-inflammatory diseases [13], cancer [14,15], and nervous system regulation [16,17]. According to the structure, ginsenosides mainly include protopanaxadiol saponin (PDS) and protopanaxatriol saponin (PTS). It has been reported that ginsenosides Re and Rg1 can inhibit LPS-induced systemic inflammation and TNBS-induced colitis in mice [18,19,20]. Although the individual ginseng ingredients have potent pharmacological effects, they are still far from clinical application and cannot be easily produced industrially. There is currently no evidence that PTS protects against DSS-induced UC in mice. Therefore, this study is focused on the therapeutic effect and protective mechanism of PTS in DSS-induced UC mice, and it is of clinical importance to find an alternative extract with better therapeutic effect.

Metabolomics is a newly developed approach after genomics and proteomics and is an important part of systems biology. It can characterize the dynamic changes in metabolites throughout the biological system triggered by natural environmental fluctuations or external perturbations. Currently, liquid chromatography–mass spectrometry (LC-MS), gas chromatography–mass spectrometry (GC-MS), and nuclear magnetic resonance (NMR) are the most commonly used techniques in screening targets in metabolomics. LC-MS is the most widely used and promising metabolomics technology because it has high resolution and is sensitive enough to be used in complex systems such as plasma and tissue [21,22]. Non-targeted metabolomics analyses reveal disease pathogenesis and potential drug mechanisms through biomarker discovery, which offers a wide range of applications in drug discovery [23,24,25]. Most likely, the identification of clinically relevant metabolites of new potential biomarkers could also help to assess prognoses and develop new treatment strategies.

We evaluated the protective effect of PTS against UC and investigated the underlying mechanism using metabolomics. These results may provide evidence to support PTS as treatment for UC.

## 2. Results

### 2.1. Determination of the Main Components of Triol Saponins

#### 2.1.1. Calibration Curves and Limits of Detection and Quantification

**Figure 1:**
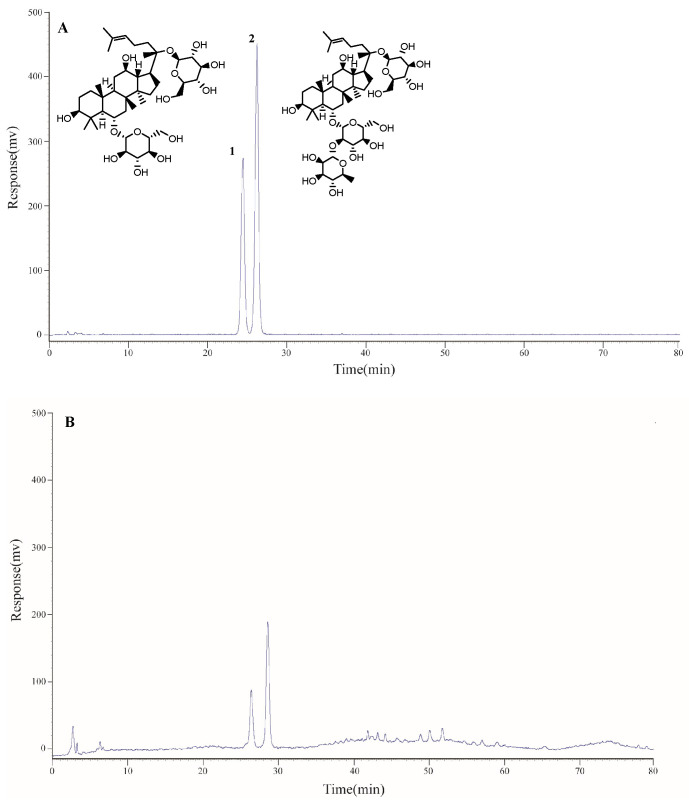
HPLC-ELSD chromatograms of mixed standards (**A**) and PTS (**B**): 1. Ginsenoside Rg1; 2. Ginsenoside Re.

The HPLC-ELSD method was created for the simultaneous determination of the two identified compounds in PTS (Figure 1). The linearity study of the HPLC-ELSD method on two compounds showed that the linearity within the test range was good (R^2^ ≥ 0.9990). The linear range and correlation coefficient of the calibration curve are shown in Table S1.

The two substances had a limit of detection (LOD) between 5.6 and 6.0 g/mL and a limit of quantification (LOQ) between 14.0 and 15.0 g/mL. These findings showed the established HPLC-ELSD method to be accurate and sensitive.

#### 2.1.2. Precision, Repeatability, Stability, and Accuracy

The studied intra-day and inter-day precisions with standard solutions are shown in the Electronic supplementary information. The relative standard deviation (RSD) values were all below 2.4%, indicating good precision of the method. The repeatability results showed that the maximum RSD was 2.5%. The RSD value of the stability test was less than 2.4% (Table S2), indicating that the PTS test solutions remained stable for at least 12 h. The overall recovery rate of these two components ranged from 99.6% to 100.5%, and the RSD was less than 2.6% (Table S2). The technique is accurate in based on the above data.

#### 2.1.3. Contents of Two Components of Triol Saponins

The above-mentioned validated analysis method was used for the analysis of PTS (n = 3). The contents of the two components in PTS are shown in Table 1.

### 2.2. Therapeutic Effect of PTS on DSS-Induced UC Mice

**Figure 2:**
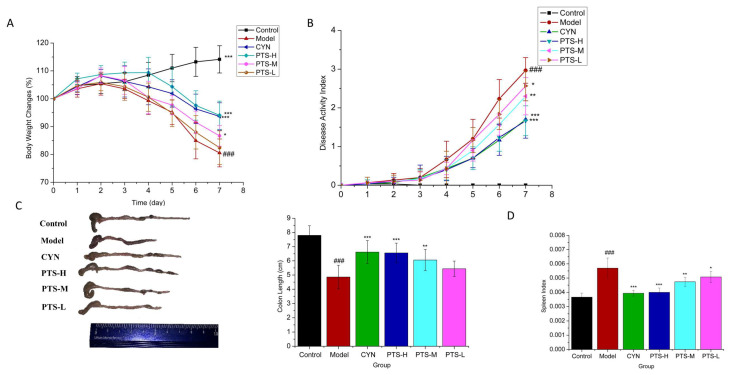
Effects of PTS on clinical symptoms of mice with DSS-induced ulcerative colitis: (**A**) body weight change of each group (n = 10); (**B**) disease activity index (DAI) (n = 10); (**C**) colon length (n = 10); (**D**) spleen index (n = 10). Data are expressed as the means ± SDs. ### *p* < 0.001 vs. control. * *p* < 0.05, ** *p* < 0.01, and *** *p* < 0.001 vs. model group.

In our investigation, we used DSS create an acute UC model in BALB/c mice. Our findings demonstrated that the weight change of mice in the model group decreased significantly and gradually compared with the control group, and the disease activity index increased significantly (*p* < 0.001) (Figure 2A,B); diarrhea and hematochezia symptoms occurred. In addition, the length of the colon was significantly shortened in the model group, and the spleen index was also significantly increased (*p* < 0.001) (Figure 2C,D). All these results indicated that we successfully established an acute UC model in mice. Moreover, PTS groups of three doses were used, including high-dosage PTS (PTS-H), middle-dosage PTS (PTS-M), and low-dosage PTS (PTS-L). The PTS treatment groups showed that treatment could significantly alleviate these abnormal changes caused by DSS, indicating that PTS caused significant improvement in acute UC, especially PTS-H and PTS-M (*p* < 0.05), and the PTS-H group showed no significant differences compared with the CYN group.

### 2.3. Results of ELISA Analysis

**Figure 3:**
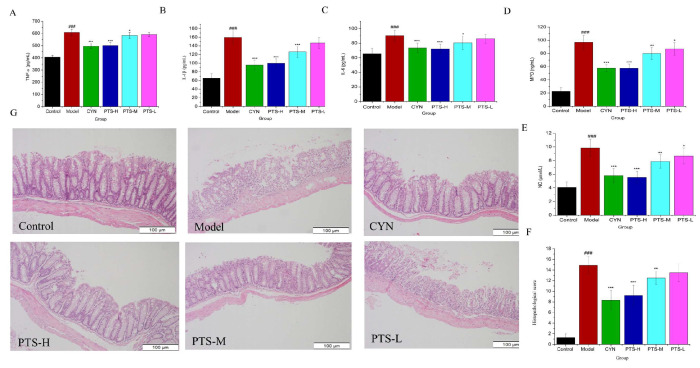
Results of the ELISA analyses: (**A**) TNF-α in serum in the six groups; (**B**) IL-6 in serum in the six groups; (**C**) IL-1β in serum in the six groups; (**D**) MPO in colon in the six groups; (**E**) NO in colon in the six groups; (**F**) histological score (n = 5); (**G**) representative images of hematoxylin and eosin (H&E)-stained colon tissues in mice of each group. Scale bar = 100 µm (n = 5). Data are expressed as the means ± SDs (n = 10). ### *p* < 0.001 vs. control. * *p* < 0.05, ** *p* < 0.01, and *** *p* < 0.001 vs. model group.

To further investigate the anti-inflammatory effects of PTS, cytokines were detected in the colon and serum of mice in each group. Our findings (Figure 3) demonstrated that compared with the normal control group, the model group’s serum levels of TNF-α, IL-6, and IL-1 were significantly higher (*p* < 0.001), and the model group had significantly increased levels of MPO and NO in the colon (*p* < 0.001), showing that the model group experienced a strong inflammatory response.

PTS significantly reduced the levels of TNF-α, IL-6, and IL-1β in serum (Figure 3A–C) and significantly decreased the levels of MPO and NO in the colon (Figure 3D,E), especially in PTS-H and PTS-M (*p* < 0.05), and the activity of the PTS-H group was basically the same as that of the CYN group. The results show that PTS has a significant anti-inflammatory effect.

### 2.4. Histopathological Evaluation

The model group’s histology score dramatically increased (*p* < 0.001) when compared with the control group (Figure 3F). The histopathological analysis of the model revealed substantial damage to the colon mucosa and the loss of a significant portion of the intestinal crypt. In the mucosal and submucosal layers, there were also many inflammatory cell infiltrations (Figure 3G). However, compared with the model group, the histological scores of the PTS-H and PTS-M treatment groups were significantly reduced (*p* < 0.01); additionally, tissue remained intact with mild edema of the intestinal crypt and just a small amount of inflammatory cell infiltration in the mucosal layer, and there were no significant differences compared with the CYN group (*p* > 0.05), demonstrating that the mucosa was significantly protected by PTS.

### 2.5. Results of Serum and Colon Tissue Metabolomics

#### 2.5.1. Validation of UPLC-QTOF-MS

The *m*/*z*-retention time (RT; min) pairs selected in the positive electrospray ionization (ESI+) mode for the system stability test included: 104.1107–0.66, 565.8848–5.11, 574.3048–11.14, 867.3590–14.47, 365.2294–16.33, 359.1978–18.27, 572.3672–18.90, and 568.5625–26.65. The *m*/*z*-RT pairs in negative electrospray ionization (ESI-) mode were as follows: 115.0041–0.66, 845.3060–5.07, 369.2265–10.17, 566.3458–18.19, 569.3568–19.17, 568.3622–20.18, 371.2190–22.87, and 359.2954–26.48.

The relative standard deviations (RSDs) of the peak intensities (PIs) and RT in the system stability, precision, reproducibility, and sample stability tests are given in Table S3.

The above-mentioned validation showed that the UPLC-QTOF-MS method was effective in terms of precision, repeatability, and stability.

The serum and colon samples were tested using the validated approach. Additionally, the representative chromatograms of the base peak intensity (BPI) for each group are displayed in Figure S1.

#### 2.5.2. Multivariate Statistical Analyses

**Figure 4:**
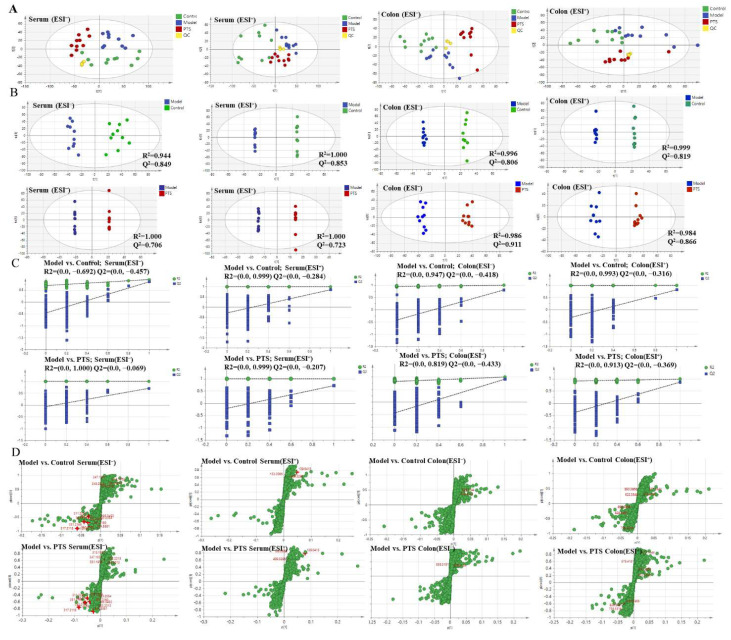
The metabolic profiles of serum and colon were used to calculate the PCA score (**A**), OPLS-DA score (**B**), permutations test plots (**C**), and OPLS-DA S-plots (**D**).

A principal component analysis (PCA) was performed on serum and colonic metabolic profiles of mice for each group (Figure 4A). The QC samples were clustered relatively to the experimental samples, and there was good repeatability. The model group, the control group, and the PTS-H group could all be easily distinguished from one another in the PCA score plot in the positive and negative ion modes, according to the analysis of the PCA data. This indicated that biochemical interference occurred in the model group and the treatment groups. In spite of this, the model group was significantly distanced from each group, and each group was highly clustered, while the PTS-H treatment group tended to be near the control group, showing that the mice’s conditions after PTS-H treatment significantly improved.

In addition, a model OPLS-DA was constructed to improve the discriminant analysis ability of the model and further determine the different components between the model group and the PTS-H treatment group. Moreover, the classification effect was significant, so the groups were separated from each other (Figure 4B), indicating that the metabolite characteristics of the mice were significantly improved by the PTS-H treatment.

In general, the OPLS-DA model has stronger predictive ability when Q2 is higher than 0.5. From the permutation plots, we could see that all blue Q2 points were lower than the original right points (Figure 4C), indicating that the original model was valid and there was no overfitting. The S-plot is a tool used to show the covariance and correlation between the metabolites and the modeled class. An S-plot was created to identify the potential metabolites (Figure 4D). The further away from the origin they were, the more significantly the points in the S-plots contributed to the clustering of the model group and the PTS-H treatment group.

#### 2.5.3. Screening and Identification of Metabolites

**Figure 5:**
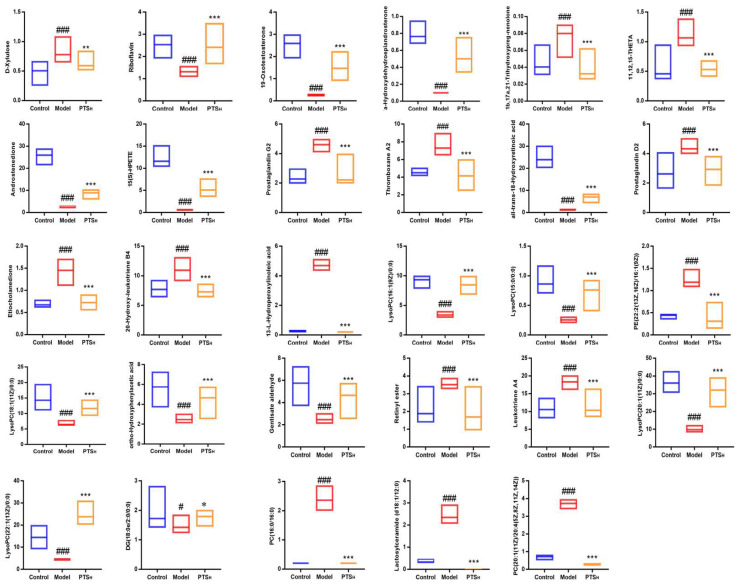
Each biomarker’s concentration in the control, model, and PTS-H groups’ serum or colon. Data are presented as means ± SDs (n = 10). Compared with control group, # *p* < 0.05 and ### *p* < 0.001; compared with model group, * *p* < 0.05, ** *p* < 0.01, and *** *p* < 0.001.

The potential biomarkers were extracted to form S-plots and perform loading plots. Only when the value of the combination variable importance in the projection, VIP, >1 in the S-plot diagram and the *p*-value < 0.05, the metabolites could be considered as potential biomarkers. Endogenous metabolites were identified based on the MS/MS fragments and online databases. On the basis of the protocols described above and the comparison of metabolites between the model group and the PTS treatment groups, 29 different biomarkers were identified (Table 2), considered to be endogenous biomarkers of UC, whose levels were significantly regulated by PTS. The levels of each biomarker in the serum and colon of the control, model and PTS-H groups are displayed in Figure 5.

**Figure 6:**
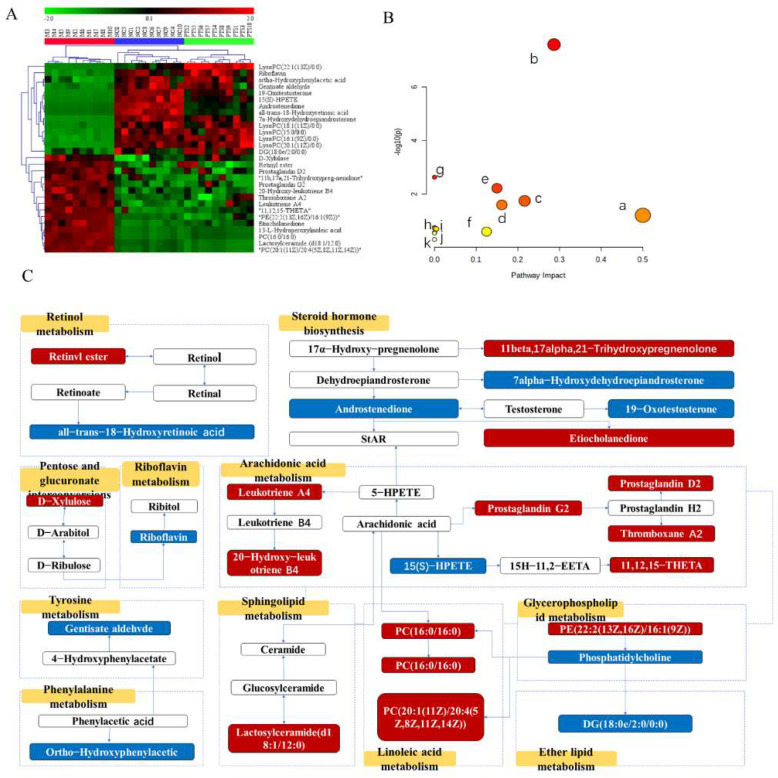
(**A**) Heatmap of biomarkers. (**B**) Effects of PTS on metabolic pathways in UC, where a, riboflavin metabolism; b, arachidonic acid metabolism; c, glycerophospholipid metabolism; d, retinol metabolism; e, steroid hormone biosynthesis; f, pentose and glucuronate interconversion; g, linoleic acid metabolism; h, phenylalanine metabolism; i, ether lipid metabolism; j, sphingolipid metabolism; k, tyrosine metabolism. (**C**) Metabolic network including biomarkers and metabolisms.

A hierarchical clustering heat map was then constructed using the identified metabolites (Figure 6A). In this heat map, the colors from red to green indicate decreasing abundance of biomarkers. To explore the potential metabolic pathways in which the level of these biomarkers was significantly regulated by PTS, 29 endogenous biomarkers were imported into Metaboanalyst to perform a pathway enrichment analysis (Table S4). The results of the analysis showed that 11 metabolic pathways were potential target metabolic pathways of PTS in the treatment of UC in mice (Figure 6B). In addition, we mapped relative metabolic pathways with reference to the KEGG database (Figure 6C). The network showed that PTS-H treatment increased the blue metabolites and downregulated the red metabolites, respectively. This clearly indicated that PTS-H could regulate the disruption of these metabolic pathways.

## 3. Discussion

UC poses a serious public health challenge worldwide; therefore, it warrants the development of novel drugs with high efficacy. The DSS-induced UC mouse model is widely used to screen drugs against ulcerative colitis. According to reports, this type of colitis is the most typical, which is very similar to the phenotypes of UC in humans, including weight loss, blood in the stool, and diarrhea [26]. More and more scientists turn to natural products, searching for effective remedies for ulcerative colitis from natural products, and curcumin [27], ginger extract [28], and aloe gel [29] have been discovered.

To the extent that we are aware, this study is the first to study the effect of PTS against ulcerative colitis. Our results show that PTS can significantly alleviate UC in mice induced by DSS, as indicated by an increase in body weight and a decrease in the disease activity index of UC mice, as well as the alleviation of pathological damage to the colon and the inhibition of the inflammatory response in UC mice. To further support the significance of PTS in anti-ulcerative colitis, future studies may expand on the mechanism of PTS or create more UC animal models (e.g., oxazolone-induced or TNBS-induced UC model).

Meanwhile, TNF-α, IL-6, and IL-1, proinflammatory cytokines, are crucial in the development of colitis [30]. NO plays an important regulatory role in the inflammatory cascade, especially in inflammatory response and signal transduction. Inhibiting the synthesis of NO can improve inflammatory symptoms [31]. Numerous studies indicate that an excess of NO may exacerbate the clinical characteristics of ulcerative colitis by directly damaging cells, damaging intestinal epithelial cells, activating neutrophils, etc. [32]. MPO is produced by neutrophils, and a high MPO level is an indicator of neutrophil aggregation [33]. A decrease in MPO activity may reduce the severity of inflammation. Our study shows that PTS has an ameliorative effect on these inflammatory factors.

In recent years, metabolomics has been increasingly used to diagnose UC because the etiology and pathogenesis of UC have not been fully understood [34]. Our metabolomics study identified twenty-nine potential biomarkers involved in eleven metabolic pathways for PTS protection. Therefore, we assume that PTS’s multi-target mechanism must be responsible for its higher pharmacological effects, as follows: ① Riboflavin metabolism. Riboflavin (RF) belongs to the group of natural compounds necessary for the proper functioning of the immune system [35]. In various diseases, such as cataract [36], diabetes [37], and cardiovascular disease [38], RF has been reported to show antioxidant and anti-inflammatory effects. In this study, the content of Riboflavin metabolites in the model group were low, and PTS increased their contents, with values tending to be near to those of the normal group. It had the highest impact in this metabolic analysis, demonstrating its significance in the cause and therapy of UC. ② Arachidonic acid metabolism. Arachidonic acid may play a significant role in cell signaling as a second messenger [39]. In this study, there were seven metabolites regulated by PTS, including prostaglandins, leukotrienes, and other inflammatory substances, which is consistent with previous findings [40,41]. Except for the fact that the content of 15(S)-HPETE decreased in the model group, the contents of other metabolites increased, while PTS significantly reversed these metabolites’ contents. ③ Glycerophospholipid metabolism. PE (22:2(13Z,16Z)/16:1(9Z)) and a number of LysoPC were identified. It was found that the content of LysoPC in the model group decreased and the content of PE (22:2(13Z,16Z)/16:1(9Z)) increased. Previous research revealed that glycerophospholipid metabolism in UC is directly related to the onset and development of inflammation [42], which is consistent with the trend in our study. ④ Retinol metabolism. Vitamin deficiency is commonly associated with IBD, and the pathogenesis is multifactorial. Active metabolites of VA include retinoic acid and 9-cis-RA [43]. Vitamin A and its metabolites (retinoic acid and all-trans-RA) are essential for the regulation of the immune system. Therefore, these metabolites may play an indispensable role in UC [44]. There are two biomarkers in this impaired metabolic pathway, retinyl ester and all-trans-18-hydroxyretinoic acid. Compared with the control group, the content of retinyl ester increased, and that of all-trans-18-hydroxyretinoic acid decreased in the model group. ⑤ Steroid metabolism. The basic factor in maintaining intestinal immune homeostasis is intestinal epithelial cells (IECs) [45,46]. The intestinal mucosa contains intestinal epithelial cells, which create a biochemical and physical barrier separating immune cells from the bacteria. In the process of maintaining IEC homeostasis, steroid metabolism plays a significant role. Studies have shown that impaired intestinal glucocorticoid production and IEC dysfunction may play a key role in the pathogenesis of UC [47,48]. We found that PTS can interfere with five metabolites of this pathway. The contents of 11b, 17a, 21-Trihydroxypreg-nenolone, and Etiocholanedione increased in the model, while the contents of other metabolites decreased, and most of the discovered metabolites of steroid hormone synthesis were correlated to sex hormones. ⑥ Pentose and glucuronate conversion. The pentose and glucuronate conversion pathway is a major xylitol metabolic pathway in mammals. In this metabolic pathway, L-xylulose is reduced to xylitol by NADP-linked xylitol dehydrogenase and then converted to D-xylulose by NAD+-linked xylitol dehydrogenase [49]. High amounts of D-xylulose were found in the serum of UC mice in the current investigation, and PTS reduced those levels. This is a new discovery suggesting that UC may cause impaired pentose and glucuronate conversion.

These are the pathways that we found PTS to have a major impact on in regulating colitis. We also found some other signaling pathways related to colitis disease. Linoleic acid, as a precursor of arachidonic acid synthesis, can also generate HPODE and HODE through the metabolic pathways of COX and LOX [50]. In this study, we found that PTS can downregulate these metabolites in the linoleic acid pathway, such as PC (16:0/16:0), 13(S)-HPODE, and PC (20:1(11Z)/20:4(5Z,8Z,11Z,14Z)), thereby exerting an anti-inflammatory effect. Phenylalanine and tyrosine are essential amino acids that are closely related to the metabolic pathways of tyrosine and phenylalanine [51]. Currently, the occurrence and development of many diseases, including IBD, are closely related to abnormal amino acid metabolism [52,53]. Gentisate aldehyde and ortho-hydroxyphenylacetic acid are metabolites of tyrosine and phenylalanine, respectively. In this experiment, the levels of gentisate aldehyde and ortho-hydroxyphenylacetic were downregulated in the UC rat model compared with normal rats, suggesting that the metabolism of these amino acids is altered in the context of UC, which may be due to a deficiency of tyrosine and phenylalanine in rats with UC. However, these metabolites were strongly adjusted following treatment with PTS, suggesting that the disturbed balance of amino acid metabolism was corrected. Numerous crucial signal transduction processes, such as cell development, differentiation, aging, and programmed cell death, are influenced by sphingolipids [54]. According to earlier research, mucosal inflammation can cause ceramide and sphingomyelin to build up [55,56], and lactosylceramides are the most important and abundant of the diosylceramides. Compared with the DSS group, PTS was able to significantly downregulate the level of lactosylceramides (d18:1/12:0) in the colon. Interestingly, we also found that the content of DG (18:0e/2:0/0:0), which is involved in ether lipid metabolism, was significantly reduced in the UC model group, and PTS could improve the situation.

## 4. Materials and Methods

### 4.1. Medical Materials and Reagents

Natural Medicine Research Center of Jilin University provided protopanaxatriol saponins (PTS; batch number 210115). Dextran sodium sulfate (DSS; MW: 36,000–50,000 Da) was acquired from MP Biomedicals company (Santa Ana, CA, USA). The Chang Yan Ning (CYN; a positive control) table was acquired from Jiangxi CONBA Chinese Medicine Co, Ltd. (Shangrao, China). Ginsenosides Re and Rg1 were acquired from Natural Medicine Research Center of Jilin University. Riboflavin, prostaglandin E2, PC (16:0/16:0), and prostaglandin D2 with purity ≥ 95% were acquired from Merck (Darmstadt, Germany). Myeloperoxidase (MPO), interleukin-1β (1L-1β), interleukin-6 (IL-6), and tumor necrosis factor-α (TNF-α) enzyme-linked immunosorbent assay kits were purchased from Hangzhou Lianke Biotechnology Co., Ltd. (Hangzhou, China). A nitric oxide detection kit and 4% paraformaldehyde solution were acquired from Biyuntian Co., Ltd. (Shanghai, China).

Methanol and acetonitrile of UPLC grade were acquired from Thermofisher Scientific (Shanghai, China). We bought deionized water from A.S. Watson Bunch Ltd. (Hong Kong, China). All other chemical solvents were of analytical grade.

### 4.2. Quantitative and Qualitative Analyses of Protopanaxatriol Saponin

To rapidly determine the contents of the main ingredients of protopanaxatriol saponin, an analytical strategy was created utilizing HPLC-ELSD for the simultaneous test of two chemical components in PTS.

The HPLC instrument included two LC-10AT VP pumps (Shimadzu, Kyoto, Japan), an AT-330 column heater, and a CS-Light Solution chromatography workstation. Chromatographic separation was performed using an A COSMOSIL 5C18-PAQ column (4.6 mm × 250 mm) at a column temperature of 35 °C. At a flow rate of 1.0 mL/min, gradient elution was carried out using water as solvent A and acetonitrile as solvent B, injecting 10 μL of samples. The following mobile-phase gradients were used for chromatographic separations: 0–10 min (21–22% B), 10–22 min (22–23% B), 22–30 min (23% B), 30–36 min (23–30% B), 36–54 min (30–35% B), 54–64 min (35% B), 64–74 min (35–50% B), and 74–80 min (50–60% B). A SEDEX 75 (SEDERE, Olivet, France) ELSD was associated to the HPLC workshop. Drift tube temperature, 45 °C; gain value, 10; nitrogen gas pressure, 3.0 bar.

Standard stock solutions of ginsenoside Re (0.28 mg/mL) and ginsenoside Rg1 (0.24 mg/mL) were prepared separately in methanol, and the two standard solutions were mixed for HPLC analyses. Aliquots of these solutions were then diluted with methanol to a range of concentrations to generate calibration curves.

After the HPLC-ELSD analysis of the calibration solutions, the linear ranges and correlation coefficients were determined. The limit of detection (LOD) and the limit of quantification (LOQ) were defined as the analyte amounts that could be detected with S/N values of 3 and 10, respectively. Intra- and inter-day variations were selected to assess the precision of the analytical method. Intra-day precision was assessed by measuring the PTS analyte in five replicates within one day. Inter-day precision was assessed by measuring it for five days in a row. The precision was assessed using the peak area logarithm’s RSD value. Repeatability: Five different PTS test solutions were used to assess repeatability, and the RSD was used to express the variations. Stability: The PTS test solution was prepared, kept at room temperature and then injected into the HPLC instrument 0, 2, 4, 8, and 12 h later. Accuracy: A recovery experiment, in which known amounts of the standards were added to a PTS test sample, was used to evaluate the accuracy of this quantification method. The spiked samples were then processed as described above. Five replicates were prepared for the assay. Average recoveries were calculated using the following formula: Recovery (%) = (amount tested − original amount)/amount spiked × 100%.

### 4.3. Animals

SPF-grade BALB/c mice (male, 18–22 g) were acquired from the animal center of Liaoning Changsheng Biotechnology Co., Ltd., (Shenyang, China) (certificate No. 20210518). the mice were provided with standard food and water. In addition, all mice were kept in animal rooms at a room temperature of 21–23 °C, humidity of 40–60% and dark–light circulation for 12 h. Institutional Animal Care and Use Committee of Jilin University School of Pharmaceutical Science approved protocols for the use of animals in research (approval number: 20210025). Ethical Principles for the Use and Care of Animals were followed during this investigation.

### 4.4. Animal Model Establishment and Treatment Procedures

BALB/c mice were randomly selected into six groups (ten mice in each group) after a one-week acclimatization period; these were designated as the control group; the model group; the CYN (500 mg/kg) treatment group; and the PTS-H (100 mg/kg), PTS-M (50 mg/kg), and PTS-L (25 mg/kg) treatment groups. The dose of the PTS treatment groups was set according to the results of the previous pre-experiment. the control group received distilled water, while the other experimental groups received 3.5% (*w*/*v*) DSS drinking water, which the mice were given freely access to for 7 days, inducing severe ulcerative colitis. All drugs were suspended in distilled water, and each treatment group was orally administered the agent at different doses at 9:00 a.m. In addition, the control group and the model group received an equivalent dose of distilled water. The intragastric dosage was 0.2 mL/10 g of body weight.

### 4.5. Disease Activity Index Score and Sample Collection

The DAI score was recorded as the average of weight loss, stool consistency, and fecal occult blood scores based on Cooper’s scoring criteria [57]. Body weight, stool consistency, and fecal occult blood status were recorded each day, and the DAI score was defined.

All mice were prohibited from ingesting any food for 12 h but were free to drink water. Serum was collected after the removal of the eyeball. Blood samples were allowed to coagulate at room temperature for 30 min and were then centrifuged at 3000 rpm at 4 °C for 10 min. The mice were then sacrificed via cervical dislocation, and their spleen and colon were collected; the spleens were weighed, and the splenic indices were calculated. The distance between the anus and ileocecal junction was measured, and parts of the colon of the mice were chosen and fixed in 4% paraformaldehyde. Then, all samples were kept in the freezer at −80 °C until the analyses.

### 4.6. Histological Examination of Colonic Tissues

A portion of the distal colon was immersed in 4% paraformaldehyde fixative solution, embedded in paraffin, cut into 5 μm sections with a microtome, completely deparaffinized, and hydrated. Sections were prepared using a hematoxylin–eosin staining solution. Following a prior study procedure [58], the histological score of the colon tissue was calculated (which mostly includes the degree of epithelial cell destruction and the degree of inflammatory infiltration).

### 4.7. Serum and Colon Tissue Cytokine Detection

The serum levels of cytokines (TNF-α, IL-1β, and IL-6) and colon tissue MPO and NO levels were determined using a commercial ELISA kit according to the manufacturer’s instructions.

### 4.8. Metabolomics Sample Preparation

A volume of 100 µL of serum was mixed in a ratio of 1:3 with pre-chilled 80% methanol by vortexing and centrifuging at 13,000 rpm for 10 min at 4 °C. The supernatant was obtained and was freeze-dried. The dried buildup was dissolved with 100 µL of 80% methanol and filtered through a syringe channel (0.22 µm) to obtain the serum sample. A volume of 10 μL of each test sample was blended to perform the serum QC test for method validation.

We took 0.1 g of colon tissue and homogenized it with 80% methanol (1000 μL). Then, according to the preparation of the above-mentioned serum test solution, a colon test solution was prepared.

### 4.9. UPLC-Q/TOF-MS Detection Conditions

A Waters ACQUITY UPLC system with a Waters Xevo G2-S Q-TOF mass spectrometer was used to perform sample testing in both positive mode and negative mode.

Conditions for the LC system: Chromatographic separation was run on a ACQUITY UPLC BEH C18 (100 mm × 2.1 mm, 1.7 μm) from Waters Corporation (Milford, MA, USA). The mobile phases consisted of 0.1% formic acid of eluent A (water) and eluent B (acetonitrile), column temperature of 31 °C, sample temperature of 16 °C, and flow rate of 0.4 mL/min. The elution conditions applied were: 0–2 min, 10% B; 2–26 min, 10–90% B; 26–28 min, 90% B; and 28–30 min, 90–10% B. Furthermore, 10% and 90% acetonitrile were used as weak and strong wash solvents, respectively.

Conditions for the MS system: A Waters Xevo G2-S Q-TOF-MS spectrometer was equipped with an electrospray ion source (ESI) operating in different modes. Capillary voltages in positive and negative modes were 2.6 kV and −2.2 kV, respectively; other operating conditions were as follows: cone voltage, 40 V; desolvation temperature, 400 °C; cone gas flow, 50 L/h; source temperature, 150 °C; and desolvation gas flow, 800 L/h. The high-energy function was 20–40 V, and the low-energy function was 6 V. Additionally, Mass Lynx data were collected continuously using the centroid. In order to assess the stability of the device during the sample test, the QC samples were simultaneously tested four times at random over the whole worklist.

### 4.10. Validation of UPLC-QTOF-MS

The applied method was validated in ESI+ and ESI− modes as detailed below.

System stability: A serum QC sample was run randomly to monitor the stability of the system. The exact *m*/*z*-RT (min) pairs of 16 ions (from different spectral regions) were monitored.

Precision: The precision was estimated by detecting five consecutive replicates of the serum QC sample in succession.

Reproducibility: The reproducibility of sample preparation was assessed by analyzing five parallel replicates of one serum sample.

Sample stability: The stability of the sample post-preparation was evaluated by detecting one serum sample settled in autosampler for 0, 4, 8, 10, and 12 h at 4 °C.

In the above investigation, the RSDs of PI and RT in ESI+ and in ESI− modes were calculated.

### 4.11. Data Processing and Multivariate Analysis

The data collected with UHPLC-Q-TOF-MS were imported into Marker Lynx XS V4.1 software for peak detection, peak matching, peak normalization, and alignment. The preprocessed data were then put into Simca-P^®^ software (version 14.1; Umetrics, Umea, Sweden) for a principal component analysis (PCA) and an orthogonal least squares discriminant analysis (OPLS-DA). To produce a reference distribution with the R2/Q2 values suggesting statistical significance, a permutation test was also carried out. Based on the predicted importance of the variables (VIP > 1) and a *t*-test (*p* < 0.05), potential biomarkers were chosen.

The exact mass charge ratio (error < 10 ppm) and secondary information were compared with metabolite databases such as Human Metabolome Database (HMDB; http://www.hmdb.ca/, accessed on 15 December 2021) and Metlin (http://metlin.scripps.edu/, accessed on 15 December 2021) to identify potential biomarkers. Hierarchical clustering heat maps were then generated. The Encyclopedia of Genes and Genomes (http://www.kegg.com/, accessed on 21 December 2021) database and MetaboAnalyst (http://www.metaboanalyst.ca/, accessed on 21 December 2021) were used to conduct a metabolic pathway analysis and create maps of the metabolic pathways.

### 4.12. Statistical Analysis

All data results were described as mean values ± standard deviations (SDs). The statistical results were obtained using a two-tailed unpaired Student’s *t*-test or a one-way analysis of variance (ANOVA), and a *p*-value < 0.05 was regarded as statistically significant.

## 5. Conclusions

This study clarifies the role of PTS in UC treatment. A systematic pharmacodynamic evaluation demonstrated the ameliorating effects of PTS on DSS-induced colitis mice for the first time. Non-targeted metabolic analyses using UPLC-QTOF/MS identified that 29 biomarkers responsible for colitis were regulated by PTS, which was closely linked to the disruption of several metabolic pathways, such as those for riboflavin metabolism, arachidonic acid metabolism, glycerophospholipid metabolism, retinol metabolism, steroid hormone biosynthesis, pentose and glucuronate interconversion, phenylalanine metabolism, ether lipid metabolism, sphingolipid metabolism, and tyrosine metabolism. The results contribute to the understanding of the pathogenesis of UC and the discovery of targets for clinical diagnosis, and they provide further evidence for using PTS as an anti-ulcerative colitis agent.

## Figures and Tables

**Table 1:** Contents (%, mean values ± SDs, n = 3) of two compounds in PTS.

Compound	Contents (%)
Ginsenoside Rg1	18.68 ± 0.64
Ginsenoside Re	29.01 ± 0.85

**Table 2:** Endogenous metabolites identified in DSS-induced ulcer colitis mouse serum and colon that were regulated by PTS.

No.	RT (min)	Metabolite	Formula	*m*/*z*	Δm (PPM)	Adducts	HMDB ID	KEGG ID	Source	Trend	
										M vs. C	PTS-H vs. M
1a	0.69	D-Xylulose	C_5_H_10_O_5_	195.0492	−9.23	[M+FA-H]^−^	HMDB01644	C00310	Serum	↑	↓
2 *	3.40	Riboflavin	C_17_H_20_N_4_O_6_	377.1444	−3.18	[M+H]^+^	HMDB00244	C00255	Colon	↓	↑
3a	8.46	19-Oxotestosterone	C_19_H_26_O_3_	347.1851	−3.74	[M+FA-H]^−^	HMDB03959	C05295	Serum	↓	↑
4a	9.05	7a-Hydroxydehydroepiandrosterone	C_19_H_28_O_3_	349.2013	−2.00	[M+FA-H]^−^	HMDB04611	C18045	Serum	↓	↑
5a	9.98	11b,17a,21-Trihydroxypreg-nenolone	C_21_H_32_O_5_	409.2213	−4.64	[M+FA-H]^−^	HMDB06760	C05489	Serum	↑	↓
6a	10.33	11,12,15-THETA	C_20_H_34_O_5_	353.2313	−5.66	[M-H]^−^	HMDB04684	C14782	Serum	↑	↓
7a	10.50	Androstenedione	C_19_H_26_O_2_	331.191	−1.51	[M+FA-H]^−^	HMDB00053	C00280	Serum	↓	↑
8a	12.01	15(S)-HPETE	C_20_H_32_O_4_	335.2218	−2.98	[M-H]^−^	HMDB04244	C05966	Serum	↓	↑
9a	12.78	Prostaglandin G2	C_20_H_32_O_6_	367.2112	−3.81	[M-H]^−^	HMDB03235	C05956	Serum	↑	↓
10a	13.03	Thromboxane A2	C_20_H_32_O_5_	351.2165	−3.42	[M-H]^−^	HMDB01452	C02198	Serum	↑	↓
11a	13.27	all-trans-18-Hydroxyretinoic acid	C_20_H_28_O_3_	315.1962	−1.27	[M-H]^−^	HMDB12452	C16679	Serum	↓	↑
12 *	13.71	Prostaglandin D2	C_20_H_32_O_5_	351.216	−4.84	[M-H]^−^	HMDB01403	C00696	Serum	↑	↓
13a	14.05	Etiocholanedione	C_19_H_28_O_2_	333.2054	3.59	[M+FA-H]^−^	HMDB03769	C03772	Serum	↑	↓
14 *	14.57	20-Hydroxy-leukotriene B4	C_20_H_32_O_5_	351.2166	−3.13	[M-H]^−^	HMDB01509	C04853	Serum	↑	↓
15a	15.07	13-L-Hydroperoxylinoleic acid	C_18_H_32_O_4_	311.223	0.64	[M-H]^−^	HMDB03871	C04717	Serum	↑	↓
16a	16.37	LysoPC (16:1(9Z)/0:0)	C_24_H_48_NO_7_P	538.3151	0.19	[M+FA-H]^−^	HMDB10383	C04230	Colon	↓	↑
17a	16.39	LysoPC (15:0/0:0)	C_23_H_48_NO_7_P	526.3144	−1.14	[M+FA-H]^−^	HMDB10381	C04230	Colon	↓	↑
18a	17.59	PE (22:2(13Z,16Z)/16:1(9Z))	C_43_H_80_NO_8_P	814.5581	−2.82	[M+FA-H]^−^	HMDB09551	C00350	Serum	↑	↓
19a	18.26	LysoPC (18:1(11Z)/0:0)	C_26_H_52_NO_7_P	522.3548	−1.15	[M+H]^+^	HMDB10385	C04230	Colon	↓	↑
20a	18.55	ortho-Hydroxyphenylacetic acid	C_8_H_8_O_3_	153.0566	1.31	[M+H]^+^	HMDB00669	C05852	Serum	↓	↑
21a	18.59	Gentisate aldehyde	C_7_H_6_O_3_	139.0413	1.65	[M+H]^+^	HMDB04062	C05585	Serum	↓	↑
22a	18.72	Retinyl ester	C_20_H_30_O_2_	301.2163	−3.32	[M-H]^−^	HMDB0003598	C02075	Serum	↑	↓
23a	19.70	Leukotriene A4	C_20_H_30_O_3_	317.2118	−1.26	[M-H]^−^	HMDB01337	C00909	Serum	↑	↓
24a	21.15	LysoPC (20:1(11Z)/0:0)	C_28_H_56_NO_7_P	550.3866	−0.18	[M+H]^+^	HMDB10391	C04230	Colon	↓	↑
25a	23.65	LysoPC (22:1(13Z)/0:0)	C_30_H_60_NO_7_P	578.4181	0.17	[M+H]^+^	HMDB10399	C04230	Colon	↓	↑
26a	25.00	DG (18:0e/2:0/0:0)	C_23_H_46_O_4_	409.3296	1.95	[M+Na]^+^	HMDB11147	C03820	Serum	↓	↑
27 *	25.05	PC (16:0/16:0)	C_40_H_80_NO_8_P	756.5516	0.26	[M+Na]^+^	HMDB00564	C00157	Colon	↑	↓
28a	25.62	Lactosylceramide (d18:1/12:0)	C_42_H_79_NO_13_	828.5467	2.78	[M+Na]^+^	HMDB04866	C01290	Colon	↑	↓
29a	26.18	PC (20:1(11Z)/20:4(5Z,8Z,11Z,14Z))	C_48_H_86_NO_8_P	858.5968	−1.75	[M+Na]^+^	HMDB08312	C00157	Colon	↑	↓

* Metabolites validated with standards. a, Metabolites confirmed with MS/MS fragments. “C” represents control group; “M” represents model group. Metabolite content in model group compared to control increased (↑) against treatment compared to model group decreased (↓) and vice versa.

## Data Availability

All the data used in this study are available within this article. Further inquiries can be directed to the authors.

## Supplementary Materials

The following supporting information can be downloaded at: https://www.mdpi.com/article/10.3390/molecules27238346/s1, Figure S1: The serum and colon samples from the Control, Model, and PTS-H groups are shown in the representative BPI chromatograms in positive (A-F) and negative (G-L) modes; Table S1: Regression equations, correlation coefficient, linear ranges, LOD and LOQ data of the two compounds; Table S2: Precision, repeatability, stability and accuracy of two compounds by HPLC-ELSD; Table S3: The RSDs (%) of PI and RT in validation tests; Table S4: The consequences from metabolic pathways.

## Sample Availability

Samples of the compounds are not available from the authors.

## Abbreviations

BPI	Base peak intensity
CYN	Changyanning tables
DAI	Disease activity index
DSS	Dextran sulfate sodium
ELISA	Enzyme-linked immunosorbent assay
ESI	Electrospray ionization
H&E	Hematoxylin and eosin stain
IL-6	Interleukin-6
IL-1β	Interleukin-1β
MPO	Myeloperoxidase
NO	Nitric oxide
OPLS-DA	Orthogonal projections to latent structures discriminant analysis
PC	Phosphatidyl-choline
PCA	Principle component analysis
PE	Phosphatidylethanolamine
PI	Peak intensities
PTS	Protopanaxatriol saponins
PTS-H	High-dosage PTS
PTS-L	Low-dosage PTS
PTS-M	Middle-dosage PTS
PBS	Phosphate-buffered saline
QC	Quality control
QTOF-MS	Quadrupole time-of-flight mass spectrometry
RSD	Relative standard deviation
RT	Retention time
SD	Standard deviation
TNF-α	Tumor necrosis factor-α
TNBS	2,4,6-Trinitro-Benzenesulfonic acid
UC	Ulcerative colitis
UPLC	Ultra-high-performance liquid chromatography
VIP	Variable importance in the projection
