# Supplementary material for: Protective Effects of Protopanaxatriol Saponins on Ulcerative Colitis in Mouse Based on UPLC-Q/TOF-MS Serum and Colon Metabolomics
Source: Molecules. 2022 Nov 30;27(23):8346. doi: 10.3390/molecules27238346 (PMC9738265; doi:10.3390/molecules27238346)
Supplement: Supplementary file 1 [file molecules-27-08346-s001.zip › Electronic supplementary information.pdf]

Electronic supplementary information

Table S1. Regression equations, correlation coefficient, linear ranges, LOD and LOQ data of the two compounds

compound	retention time(min)	calibration curves	R^2	linear rang(μg/ml)	LOD (μg/mL)	LOQ (μg/mL)
Ginsenoside Rg1	26.398	Y=1.1317X+2.7718	0.9996	48.0-480.0	6.0	15.0
Ginsenoside Re	28.598	Y=1.2662X+2.4074	0.9990	56.0-560.0	5.6	14.0

Table S2. Precision, repeatability, stability and accuracy of two compounds by HPLC-ELSD

Compound	Precision (RSD, %)		Repeatability (RSD, %)	Stability (RSD, %)	Accuracy (n=5)	
	Intraday (n = 5)	Interday (n = 5)			Mean	RSD, %
Ginsenoside Rg1	1.8	2.1	2.5	2.3	99.6	2.3
Ginsenoside Re	2.1	2.4	2.2	2.4	100.5	2.6

Table S3. The RSDs (%) of PI and RT in validation tests

Tests	ESI^+ mode		ESI^- mode	
	PI	RT	PI	RT
system stability	1.38~3.68	0.32~1.25	1.49~3.96	0.24~1.82
precision	0.84~3.38	0.12~0.26	1.64~3.32	0.16~0.54
reproducibility	1.28~3.56	0.15~1.48	0.68~3.58	0.08~0.62
Sample stablilty	1.35~3.14	0.06~0.45	1.94~3.21	0.15~0.89

The abovementioned validation showed that the UPLC-QTOF-MS method was effective in terms of precision, repeatability, and stability.

Table S4. The consequences from metabolic pathways

Pathway Name	Match Status	p	-log (p)	Holm p	FDR	Impact
Riboflavin metabolism	1/4	0.0623	1.2055	1	0.8722	0.50
Arachidonic acid metabolism	7/36	2.64E-08	7.5783	2.22E-06	2.22E-06	0.29
Glycerophospholipid metabolism	6/36	0.0180	1.7450	1	0.3777	0.22
Retinol metabolism	2/16	0.0255	1.5935	1	0.4283	0.16
Steroid hormone biosynthesis	5/77	0.0061	2.2173	0.4971	0.1698	0.15
Pentose and glucuronate interconversions	1/18	0.2524	0.5980	1	1	0.13
Linoleic acid metabolism	3/5	0.0024	2.6262	0.1963	0.0993	0
Phenylalanine metabolism	1/12	0.1759	0.7547	1	1	0
Ether lipid metabolism	1/20	0.2763	0.5587	1	1	0
Sphingolipid metabolism	1/21	0.2880	0.5407	1	1	0
Tyrosine metabolism	1/42	0.4955	0.3050	1	1	0

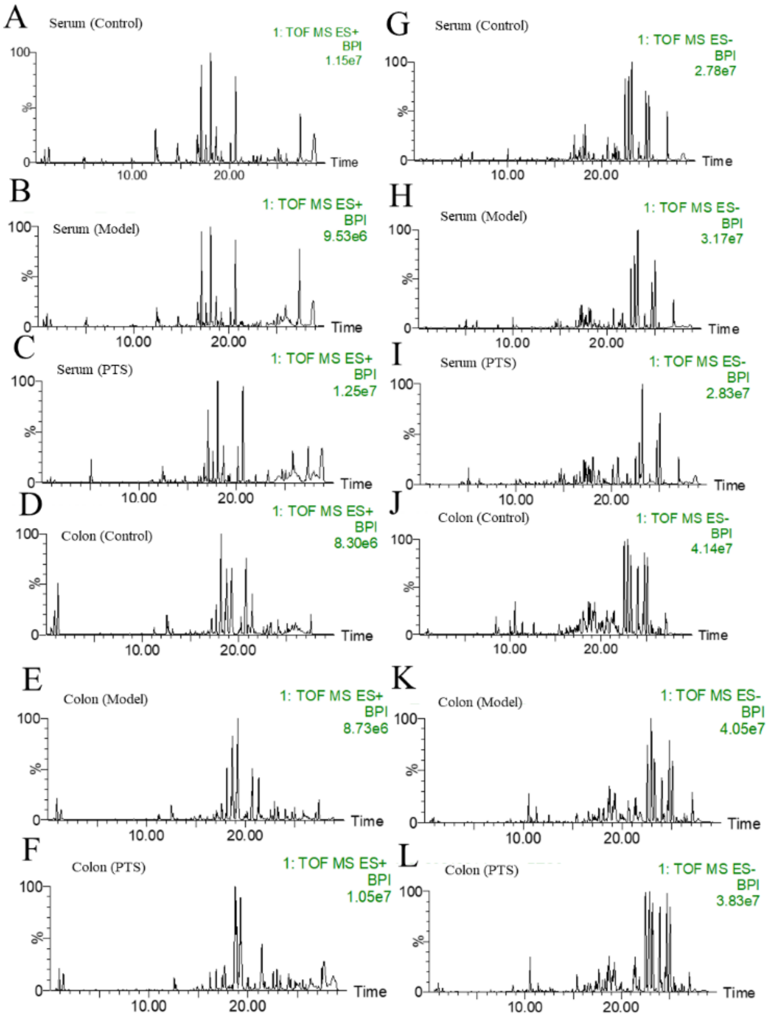

Figure S1. The serum and colon samples from the Control, Model, and PTS-H groups are shown in the representative BPI chromatograms in positive (A-F) and negative (G-L) modes.
